# Melatonin biosynthesis requires *N*-acetylserotonin methyltransferase activity of caffeic acid *O*-methyltransferase in rice

**DOI:** 10.1093/jxb/erv396

**Published:** 2015-08-14

**Authors:** Yeong Byeon, Geun-Hee Choi, Hyoung Yool Lee, Kyoungwhan Back

**Affiliations:** Department of Biotechnology, Bioenergy Research Center, College of Agriculture and Life Sciences, Chonnam National University, Gwangju, South Korea

**Keywords:** *N*-Acetylserotonin *O*-methyltransferase, caffeic acid *O*-methyltransferase, melatonin, transgenic rice.

## Abstract

Caffeic acid *O*-methyltransferase (COMT) methylates *N*-acetylserotonin into melatonin; that is, it has *N*-acetylserotonin *O*-methyltransferase (ASMT) activity. The ASMT activity of COMT was first detected in *Arabidopsis thaliana* COMT (AtCOMT). To confirm the involvement of COMT on melatonin synthesis in other plant species, the ASMT activity of a COMT from rice (*Oryza sativa*) (OsCOMT) was evaluated. Purified recombinant OsCOMT protein from *Escherichia coli* was used to validate the high ASMT activity of OsCOMT, similar to that of AtCOMT. The *K*_m_ and *V*_max_ values for the ASMT activity of OsCOMT were 243 µM and 2400 pmol min^−1^ mg protein^−1^, which were similar to those of AtCOMT. Similar to AtCOMT, OsCOMT was localized in the cytoplasm. *In vitro* ASMT activity was significantly inhibited by either caffeic acid or quercetin in a dose-dependent manner. Analogously, *in vivo* production of melatonin was significantly inhibited by quercetin in 4-week-old detached rice leaves. Lastly, the transgenic rice plants overexpressing rice *COMT* showed an increase in melatonin levels whereas transgenic rice plants suppressing the rice *COMT* had a significant decrease on melatonin levels, suggestive of the direct role of *COMT* in melatonin biosynthesis in plants.

## Highlight

Transgenic rice plants either overexpressing or suppressing rice endogenous caffeic acid *O*-methyltransferase (COMT) resulted in an increase or a decrease, respectively, in melatonin levels, suggesting a direct role of COMT on *in vivo* melatonin synthesis.

## Introduction

Melatonin (*N*-acetyl-5-methoxytryptamine) has been characterized as an important bioactive molecule that is not only implicated in animal hormones, but also in plant growth and development (Janas and Posmyk, 2013; Arnao and Hernández-Ruiz, 2014; Cipolla-Neto *et al.*, 2014; García *et al.*, 2014; Janjetovic *et al.*, 2014; Luchetti *et al.*, 2014; Hardeland, 2015; Li *et al.*, 2015; Zhang *et al.*, 2015; Zhao *et al.*, 2015). Although significant advances in elucidating the physiological roles and biochemical pathways of melatonin in animals have been achieved, studies on melatonin in plants are in their infancy, but advancing rapidly (Tan, 2015). Since melatonin was first identified from various plant species in 1995 (Dubbels *et al.*, 1995; Hattori *et al.*, 1995), almost all plant species including angiosperms and gymnosperms have been shown to produce melatonin at various levels ranging from picograms to micrograms per gram of tissue weight (Stürtz *et al.*, 2011; Zohar *et al.*, 2011; Park *et al.*, 2014; Tan *et al.*, 2014). In addition to data on melatonin contents in plants, the melatonin biosynthetic pathway and corresponding genes were identified recently (Arnao and Hernández-Ruiz, 2014; Hardeland, 2015). The key genes include tryptophan decarboxylase (*TDC*), tryptamine 5-hydroxylase (*T5H*), serotonin *N*-acetyltransferase (*SNAT*), and *N*-acetylserotonin *O*-methyltransferase (*ASMT*). Among these four genes, SNAT and ASMT play pivotal roles in regulating the relative melatonin levels in plants, because their catalytic activities are very low compared with those of TDC and T5H (Kang *et al.*, 2007; Park *et al.*, 2013a; Park *et al.*, 2013b; Byeon *et al.*, 2014b; Lee *et al.*, 2014). For example, the catalytic activity (*V*_max_/*K*_m_) of TDC was more than three and five orders of magnitude higher than those of SNAT and ASMT, respectively. Due to the low ASMT catalytic activity, transgenic rice plants overexpressing three rice *ASMT* genes showed marginal increases in melatonin levels, even in the exogenous treatment of 1mM *N*-acetylserotonin (Park *et al.*, 2013a).

Apart from ASMT, it was reported that *Arabidopsis thaliana* caffeic acid *O*-methyltransferase (AtCOMT), which is a multifunctional enzyme responsible for the production of lignin and flavonoid, can catalyse *N*-acetylserotonin into melatonin (Byeon *et al.*, 2014a; Lee *et al.*, 2014), suggestive of alternative melatonin pathways in plants. Interestingly, the catalytic activity of AtCOMT in melatonin synthesis is more than 700-fold higher than that of rice ASMT at 30 °C (Byeon *et al.*, 2014a). Based on previous observations of this AtCOMT catalytic activity, it would be interesting to explore whether COMT from other plant species also exhibits characteristics similar to those of *Arabidopsis* COMT (Nakatsubo *et al.*, 2008).

In this study, a rice (*Oryza sativa*) *COMT* (*OsCOMT*) gene was expressed in *Escherichia coli*, and the resulting purified recombinant OsCOMT showed high ASMT activity, similar to that of purified recombinant AtCOMT. The ASMT activity of OsCOMT was strongly inhibited by other COMT substrates such as caffeic acid and quercetin. Finally, melatonin production was significantly reduced or increased when the rice plants overexpressed or suppressed the endogenous rice COMT, suggestive of actual involvement of COMT in plant melatonin biosynthesis.

## Materials and methods

### Vector construction and *E. coli* expression

Full-length rice *OsCOMT* cDNA (GenBank accession number; AK064768) was kindly provided by the RIKEN BioResource Center (Kikuchi *et al.*, 2003). Full-length *OsCOMT* was amplified by PCR using a specific primer set. The forward and reverse primers were 5′-AAA AAG CAG GCT CCA TGG GTT CTA CAG CCG C-3′ and 5′-AGA AAG CTG GGT CTA CTT TGT GAA CTC-3′, respectively. The resulting PCR product was further amplified using a primer set harbouring the *attB* recombination sequences (forward primer, 5′-GGG GAC AAG TTT GTA CAA AAA AGC AGG CT-3′; reverse primer, 5′-GGG GAC CAC TTT GTA CAA GAA AGC TGG GT-3′). The resulting products were gel-purified and cloned into the pDONR221 Gateway® vector (Invitrogen, Carlsbad, CA, USA) using the BP recombination reaction. The pDONR221:OsCOMT construct was then recombined with the pET300 Gateway destination vector through LR recombination to generate pET300-OsCOMT, followed by transformation into *E. coli* BL21 (DE3) (Invitrogen). Cell culture and affinity purification steps using a Ni-NTA column were performed according to the manufacturer’s instructions (Qiagen, Tokyo, Japan). Purified recombinant OsCOMT protein was concentrated using an Ultrafree-4 centrifugal filter (Biomax-10, Millipore, Bedford, MA, USA), dissolved in 10mM Tris-HCl (pH 8.0) containing 50% glycerol, and stored at −20 °C until further analysis.

### Measurement of ASMT activity

Purified recombinant OsCOMT proteins were incubated in (a total volume of 100 µl) 100mM potassium phosphate buffer (pH 7.8) containing 1mM *N*-acetylserotonin (NAS) and 0.5mM *S*-adenosyl-l-methionine at 37 °C (or varying temperatures) for 1h and terminated by the addition of 50 µl methanol. A 10-µl aliquot was subjected to HPLC with a fluorescence detector system (Waters, Milford, MA, USA). The samples were separated on a Sunfire C18 column (Waters; 4.6×150mm) using the following gradient elution profile: from 42% to 50% methanol in 0.1% formic acid for 27min, followed by isocratic elution with 50% methanol in 0.1% formic acid for 18min at a flow rate of 0.15ml min^−1^. Melatonin was detected at 280nm (excitation) and 348nm (emission). All measurements were reproduced in triplicate. The protein concentration was determined using the Bradford method with a protein assay dye (Bio-Rad, Hercules, CA, USA). The effects of caffeic acid and quercetin on ASMT activity were examined using various concentrations of caffeic acid and quercetin, respectively. The substrate affinity (*K*_m_) and maximum reaction rate (*V*_max_) values were calculated from Lineweaver—Burk plots.

### Subcellular localization of OsCOMT

The pER-mCherry vector (a kind gift from Dr H.G. Kang, Texas State University, San Marcos, TX, USA) was used for subcellular localization analysis of OsCOMT, as described previously (Byeon *et al.*, 2014b). Briefly, full-length *OsCOMT* cDNA was PCR-amplified using a primer set containing an *Asc*I site (forward 5′-GGG GGC GCG CCA TGG GTT CTA CAG CCG C-3ʹ; reverse 5ʹ-GGG GGC GCG CCG CTT TGT GAA CTC GAT GGC-3ʹ). The resulting PCR products were gel-purified and ligated into the T&A vector (T&A-OsCOMT; RBC Bioscience, New Taipei City, Taiwan). The *Asc*I insert of *OsCOMT* from T&A-OsCOMT was ligated into the *Asc*I site of the binary vector pER8-mCherry to generate pER8-OsCOMT:mCherry. The plasmid was transformed into the *Agrobacterium tumefaciens* strain GV2260 using the freeze-thaw method, and transient expression analyses were performed as described by Voinnet *et al.* (2003). Briefly, 2-week-old tobacco (*Nicotiana benthamiana*) leaves were infiltrated with *Agrobacterium* strains followed by infiltration with 10 µM β-estradiol for transgene induction, as described previously (Byeon *et al.*, 2014b). Images were generated using a Leica TCS-SP5 confocal microscope (Leica Microsystems, Exton, PA, USA). OsCOMT:mCherry was excited using an orange He-Ne laser (594nm), and the emitted light was measured at 576–629nm. Chloroplasts were excited using a blue argon laser (488nm), and emitted light was collected at 660–731nm. Individual signals were later superimposed.

### Effects of caffeic acid and quercetin on melatonin synthesis in rice plants

Detached leaves of 4-week-old rice plants grown in a growth room were transferred into a 50-ml polypropylene conical tube containing 15ml water supplemented with 100 µM caffeic acid or quercetin for 1 d, and then further incubated for 3 d in the presence of 0.2mM cadmium for inducing melatonin synthesis (Byeon *et al.*, 2015b) in a plant growth room at 28 °C under a 16-h light/8-h dark cycle at a photosynthetic photon flux density (PPFD) of 150 μmol m^–2^ s^–1^. To quantify melatonin content, rice leaves (0.1g) were ground using a TissueLyser II (Qiagen) and extracted with 1ml chloroform. The chloroform extract (0.1ml) was evaporated to dryness and dissolved in 0.1ml 40% methanol (MeOH). Aliquots (10 µl) were analysed using an HPLC system equipped with a fluorescence detector system (2475; Waters), as described above. All measurements were reproduced in triplicate.

### Transgenic rice plant generation

A pIPKb002 binary vector was used to obtain transgenic rice plants expressing the rice *COMT* gene constitutively as described previously (Byeon *et al.*, 2015a). The pDONR221-OsCOMT gene entry vector was recombined with the pIPKb002 Gateway destination vector via LR recombination to form pIPKb002-OsCOMT. As for RNAi suppression system, pTCK303 binary vector (Wang *et al.*, 2004) was employed for downregulating an endogenous rice *COMT* gene (a kind gift from Dr Kang Chong of the Institute of Botany, Chinese Academy of Sciences, Beijing, China). A 430-bp *COMT* cDNA fragment was amplified by PCR with the primer set as follows: forward 5′-CC*A CTA GT*A TGG GTT CTA CAG CCG-3′ (*Spe*I site underlined) and reverse 5′-TCA *GAG CTC* TCC ATG AGG AC-3′ (*Sac*I site underlined). The resulting product was cloned into the T&A cloning vector (T&A:OsCOMT; RBC Bioscience), and then the *COMT* insert (antisense *COMT*) was digested by *Sac*I and *Spe*I restriction enzymes and ligated into the pTCK303 vector which was predigested by the same restriction enzymes. Thereafter, a *Kpn*I and *Bam*HI *COMT* insert (sense *COMT*) was gel-purified from the T&A:OsCOMT plasmid after digestion with *Kpn*I and *Bam*HI. The *Kpn*I and *Bam*HI *COMT* insert was further ligated into the above pTCK303 harbouring the antisense *COMT* which was predigested with *Kpn*I and *Bam*HI. The pIPKb002-OsCOMT and pTCK303:OsCOMT RNAi binary vector were transformed into *Agrobacterium tumefaciens* LBA4404 strain and followed by rice transformation as described previously (Byeon *et al.*, 2015a).

### Statistical analysis

Paired *t*-tests were performed using SigmaPlot version 10 software (Systat Software, Point Richmond, CA, USA). *P*-values <0.05 indicated statistical significance.

## Results

### Cloning and enzyme kinetic analysis of the purified recombinant rice COMT

**Fig. 1.**
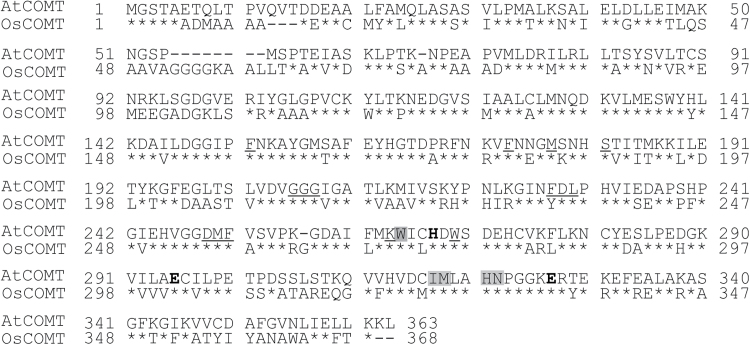
Amino acid sequence comparisons between AtCOMT and OsCOMT. The SAM-binding sites are underlined and the phenolic substrate-binding sites shaded. The three catalytic residues of COMT (His267, Glu295, and Glu327) are shown in bold letters.

A prior study by the authors showed that, in addition to its known COMT activity, AtCOMT can catalyse NAS into melatonin (Byeon *et al.*, 2014a). To determine whether plant COMTs other than *Arabidopsis* have the same ability to synthesize melatonin from the substrate NAS, a rice COMT (OsCOMT), which catalyses various phenolic and flavonoid substrates into the corresponding *O*-methylated products (Lin *et al.*, 2006; Koshiba *et al.*, 2013) was examined. However, no *O*-methylated products (such as melatonin) have been reported to be generated by OsCOMT. *AtCOMT* as the query sequence was searched against the rice genome using the BLAST database, and only one orthologous *COMT* gene was identified. The OsCOMT protein shares 62% identity with AtCOMT (Fig. 1). As expected, *S*-adenosyl-l-methionine (SAM)-binding sites, catalytic sites, and phenolic substrate-binding residues were well conserved between OsCOMT and AtCOMT. Both COMTs showed identical catalytic and substrate-binding sites, as well as identical SAM-binding sites, excluding one amino acid difference, Tyr234, in OsCOMT instead of the Phe228 in AtCOMT.

**Fig. 2.**
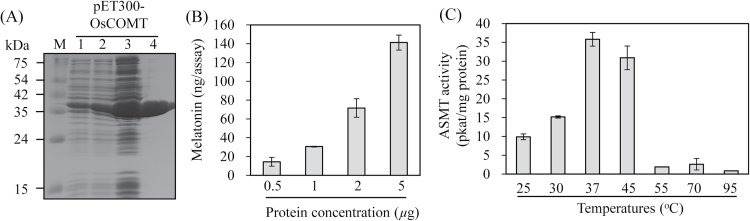
Purification of recombinant OsCOMT protein and enzyme activity. (A) Purification of N-terminal 6× histidine-tagged OsCOMT. ASMT activity of purified OsCOMT according to (B) enzyme concentration and (C) temperature. *E. coli* harbouring pET300-OsCOMT was incubated with 1mM IPTG (isopropyl-*β*-d-thiogalactopyranoside) at 28 °C for 5h. Protein samples were separated by SDS-PAGE and stained with Coomassie blue. M, molecular mass standards; lane 1, total proteins in 15-µl aliquots of bacterial culture without IPTG; lane 2, total proteins in 15-µl aliquots of bacterial culture with IPTG; lane 3, 20 µg soluble protein; lane 4, 10 µg OsCOMT purified by affinity chromatography. *In vitro* melatonin production was measured at 37 °C or varying temperatures using purified N-terminal 6× histidine-tagged OsCOMT. These data represent the mean ± standard deviation of triplicate experiments.

To explore whether OsCOMT exhibits melatonin synthesis activity via methylation of NAS [alias *N*-acetylserotonin *O*-methyltransferase (ASMT) activity] as a substrate, an N-terminal 6× histidine-tagged OsCOMT was purified, giving rise to an approximately 2mg protein yield from 100ml *E. coli* culture (Fig. 2A). The effects of protein concentration and temperature on the ASMT activity of purified recombinant OsCOMT are shown in Fig. 2B, C. The ASMT activity increased with protein concentration and temperature, with optimum activity seen at 37 °C. However, ASMT activity at 55 °C was completely inhibited. These data for OsCOMT were similar to those found for AtCOMT (Byeon *et al.*, 2014a).

**Fig. 3.**
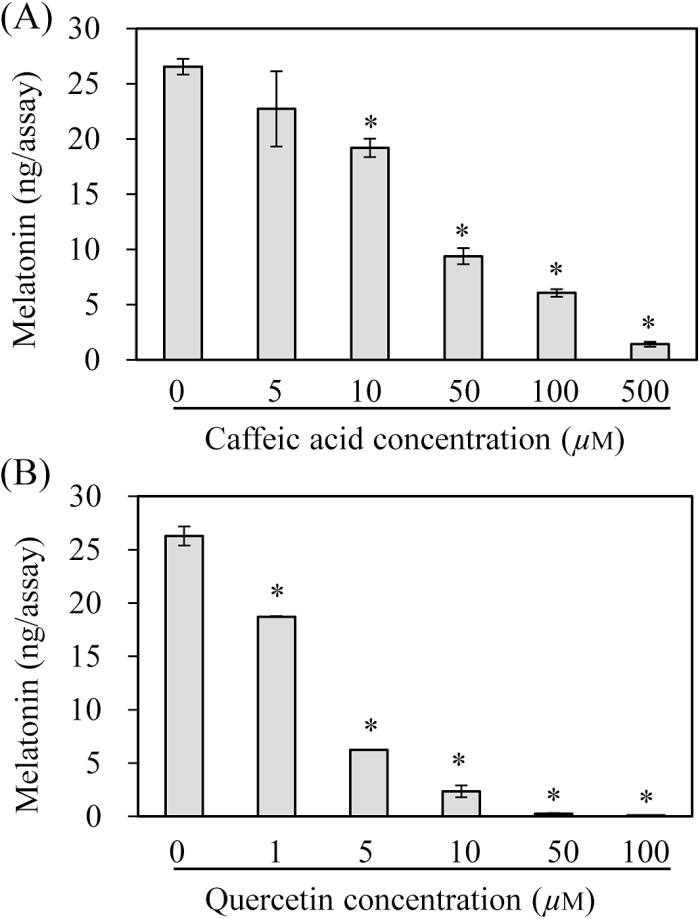
Effects of various substrates on ASMT activity. (A) Inhibition of ASMT activity by caffeic acid and (B) quercetin. ASMT activity assays were performed in the presence of various concentrations of either caffeic acid or quercetin. ASMT activity assays for OsCOMT were performed in the presence of 0.5mM NAS and various concentrations of caffeic acid or quercetin. Asterisks (*) indicate a significant difference from the control (*P*<0.05).

Owing to the multiple substrate acceptance of the COMT enzyme, it was shown that one substrate acts as a competitive inhibitor of the catalysis of another substrate. To examine whether ASMT activity is inhibited by either phenolic or flavonoid substrates, ASMT activity was measured in the presence of either caffeic acid or quercetin (Fig. 3). The ASMT activity significantly decreased as the caffeic acid concentration increased. For example, the ASMT activity after the addition of 10 µM caffeic acid resulted in 73% ASMT activity compared with the untreated control. In the presence of 100 µM caffeic acid, the ASMT activity decreased to 23% relative to the control (0 µM caffeic acid). Strikingly, quercetin treatment induced a more significant inhibition of ASMT activity. For example, the ASMT activity decreased to 23% even in the presence of 5 µM quercetin compared with the control, suggesting that quercetin exhibits a 20-fold higher inhibitory effect on ASMT activity compared with caffeic acid. The ASMT activity was completely abolished in the presence of 50 µM quercetin treatment. These data suggest that the ASMT activity of OsCOMT may be dependent on the contents of caffeic acid and quercetin in plant cells or among plant species.

**Fig. 4.**
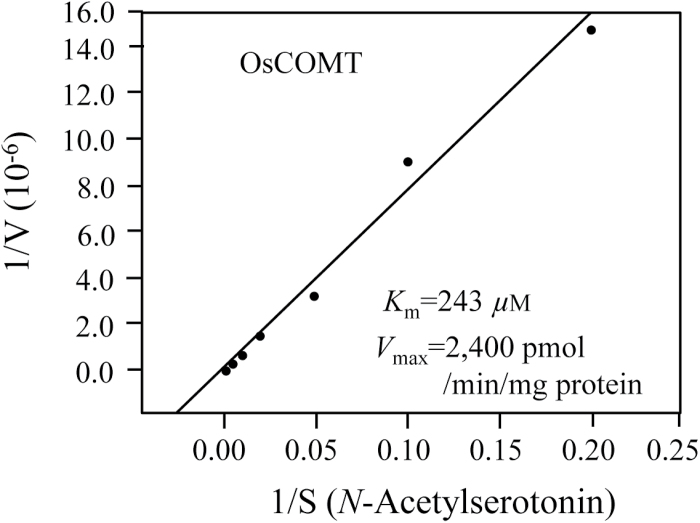
Measurement of the *K*_m_ and *V*_max_ of OsCOMT for NAS. OsCOMT (1 µg) was incubated with different concentrations of substrate for 30min at 37 °C. The *K*_m_ and *V*_max_ values were determined using Lineweaver–Burk plots.

The Michaelis–Menten plot for OsCOMT is shown in Fig. 4. The *K*_m_ and *V*_max_ values for ASMT activity using NAS as a substrate were 243 µM and 2.4 nmol min^−1^ mg protein^−1^, respectively, which were very similar to those reported for AtCOMT (Byeon *et al.*, 2014a). The kinetic data for OsCOMT suggest that the COMT proteins of almost all plant species (Lam *et al.*, 2007) have ASMT activity and play a role in melatonin production.

### Subcellular localization of rice COMT

**Fig. 5.**
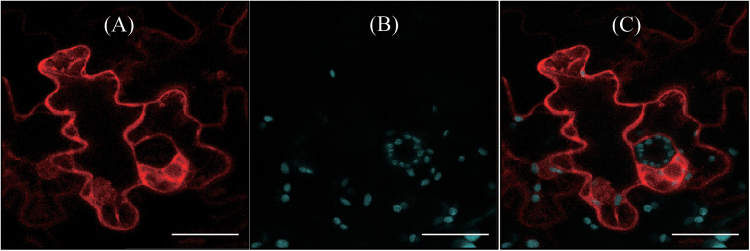
Localization of OsCOMT. (A) Red fluorescence of OsCOMT-mCherry and (B) chlorophyll (Chl) autofluorescence. (C) The two fluorescence images were merged (A+B). Tobacco (*N*. *benthamiana*) leaves were infiltrated with *Agrobacterium* harbouring the XVE-inducible OsCOMT-mCherry binary vector, as described in Materials and methods. Bars, 40 μm. (This figure is available in colour at *JXB* online.)

Analogous to AtCOMT, OsCOMT does not appear to contain any leader sequence that targets the protein into cellular compartments other than the cytoplasm. This lack of leader or transit sequences suggests that OsCOMT is localized in the cytoplasm. To support this hypothesis, the localization of OsCOMT was investigated using mCherry marker. As shown in Fig. 5, confocal microscopy clearly revealed the red fluorescence of OsCOMT-mCherry in the cytoplasm, similar to AtCOMT (Byeon *et al.*, 2014a). These fluorescence patterns do not overlap with chlorophyll autofluorescence, confirming that OsCOMT is not translocated into other subcellular compartments such as chloroplasts, as predicted based on the primary sequence information of OsCOMT.

### Inhibition of melatonin synthesis by flavonoid quercetin in the detached rice leaves

**Fig. 6.**
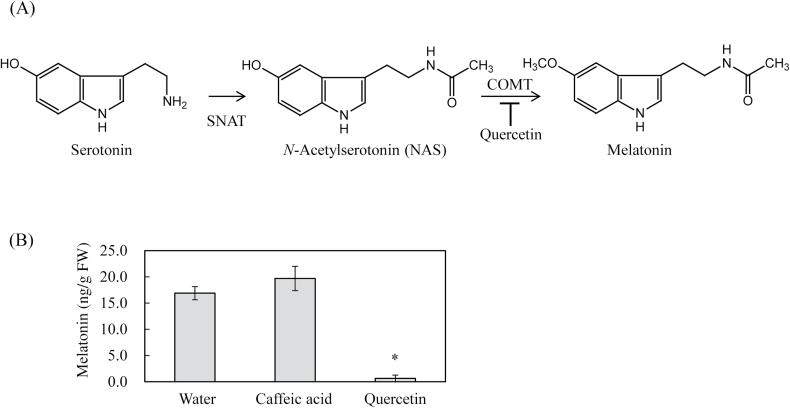
Melatonin quantification in the detached rice leaves upon cadmium treatment. (A) Proposed melatonin biosynthesis in rice via the COMT enzyme, which is inhibited by quercetin. (B) Melatonin contents in response to caffeic acid or quercetin. The detached leaves of 4-week-old rice plants were transferred into a 50-ml polypropylene conical tube containing 15ml water with either 0.1mM caffeic acid or 0.1mM quercetin and incubated for 1 d. The samples were then treated with 0.2mM cadmium for inducing melatonin synthesis for 3 d at 28 °C under a 16-h light/8-h dark cycle. The data represent the means ± standard errors of three replicates. FW: fresh weight. Asterisks (*) indicate a significant difference from the control (*P*<0.05).

Next, it was examined whether the *in vitro* inhibitory effects of caffeic acid and quercetin on ASMT activity are closely associated with the inhibition of *in vivo* melatonin synthesis. To this end, detached rice leaves were used, which were first treated with either caffeic acid (0.1mM) or quercetin (0.1mM) for 24h, followed by cadmium treatment (0.2mM) for 3 d, since cadmium induces melatonin synthesis in detached rice leaves (Byeon *et al.*, 2015b). As shown in Fig. 6, cadmium treatment induced melatonin up to 18ng g^−1^ fresh weight (FW). Quercetin pretreatment abolished melatonin production, whereas caffeic acid showed no inhibitory effect on melatonin production, indicating that quercetin is actively involved in the inhibition of melatonin production *in vivo*, and that COMT itself positively plays a role in melatonin production in plants upon cadmium treatment.

### Characterization of transgenic rice plants with overexpression or suppression of rice COMT

**Fig. 7.**
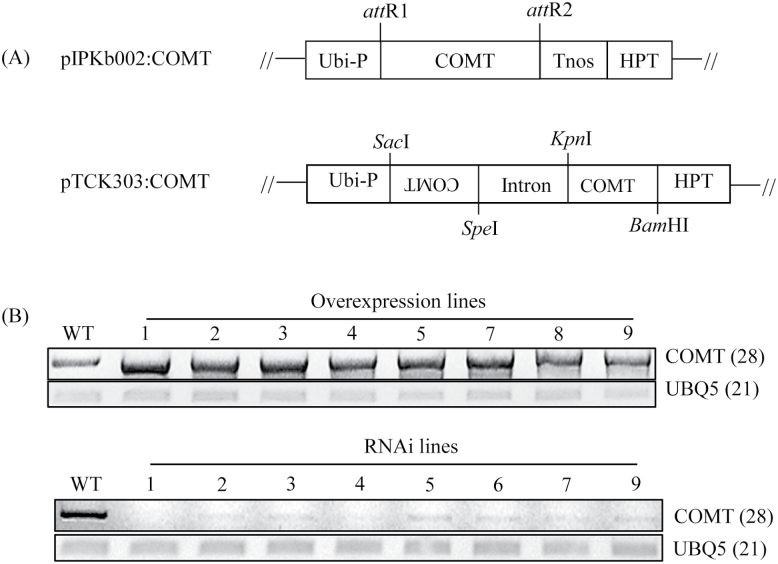
Binary vector structure and generation of *COMT* overexpression and suppression transgenic rice plants. (A) Schematic diagram of binary vectors of pIPKb002:OsCOMT and pTCK303:OsCOMT. (B) Flag leaves (T_0_) used for total RNA isolation and RT-PCR analysis of wild-type and transgenic lines. Eight independent transgenic lines were generated and grown in a paddy field. The numbers in parentheses indicate the number of PCR cycles. Ubi-P, maize ubiquitin promoter; Tnos, nopaline synthase terminator; HPT, hygromycin phosphotransferase; WT, wild-type. UBQ5, rice ubiquitin5 gene.

**Fig. 8.**
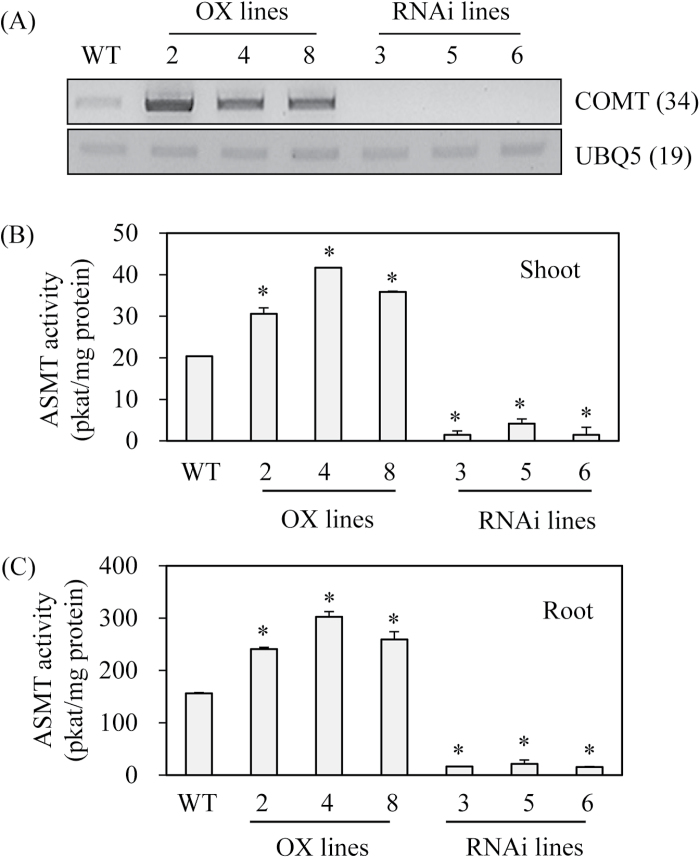
Expression levels of *COMT* transcript and ASMT enzyme activity. (A) RT-PCR analysis of *COMT* mRNA in wild-type and transgenic lines. (B) ASMT enzyme activity in the shoot of wild-type and transgenic lines. (C) ASMT enzyme activity in the root of wild-type and transgenic lines. Seven-day-old seedlings grown in a half strength MS medium were employed. The numbers in parentheses indicate the number of PCR cycles used. Asterisks (*) indicate significant differences from the wild-type (*P*<0.05). WT, wild-type; OX lines, *COMT*-overexpressed transgenic lines (T2); RNAi lines, *COMT*-suppressed RNAi transgenic lines (T2).

**Fig. 9.**
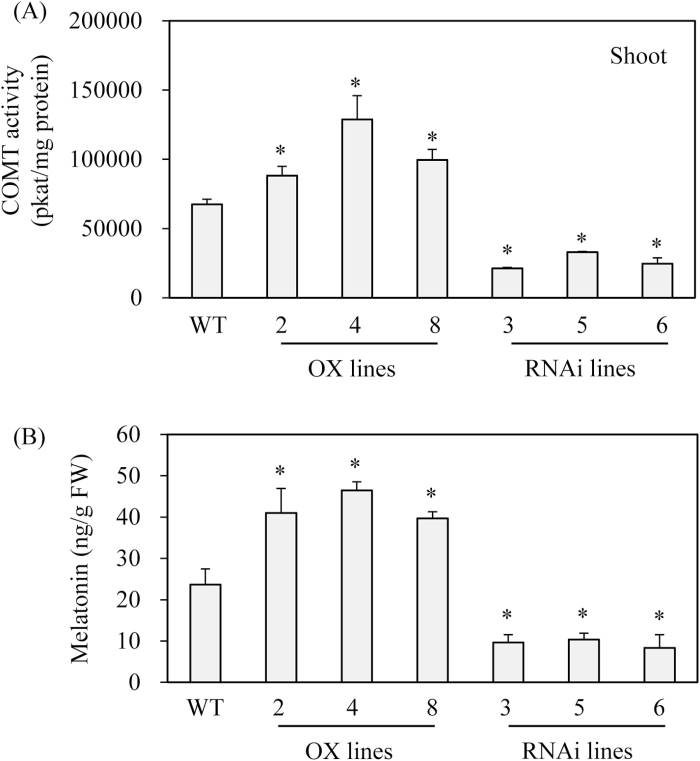
COMT enzyme activity and melatonin levels in wild-type and transgenic lines (T2). (A) COMT enzyme activity measurements in wild-type and transgenic lines. (B) Melatonin levels in wild-type and transgenic lines. Shoot of 7-d old seedlings were employed for COMT enzyme activity. As for melatonin analysis, 1-month-old rice leaves were detached and challenged with 0.2mM cadmium for 3 d. Asterisks (*) indicate significant differences from the wild-type (*P*<0.05). WT, wild-type; OX lines, *COMT*-overexpressed transgenic lines; RNAi lines, *COMT*-suppressed RNAi transgenic lines.

To examine the direct role of *COMT* in melatonin biosynthesis, transgenic rice plants with either overexpression or suppression of rice *COMT* were generated. A total of eight independent T_0_ transgenic lines were generated for each condition for further analysis (Fig. 7). All eight overexpression lines (T_0_) constitutively overexpressed rice *COMT*, while all eight RNAi suppression lines (T_0_) showed markedly downregulated expression of *COMT* mRNA. From these T_1_ seeds, T_2_ lines with three independent lines were further selected. These T_2_ lines had *COMT* expression patterns identical with those observed in T_0_ lines (Fig. 8A). Similar to the *COMT* mRNA expression patterns, ASMT enzyme activity was increased by an average of 1.7-fold in the overexpression lines, whereas ASMT enzyme activity was decreased by eight-fold in the RNAi lines relative to the wild-type controls (Fig. 8B, C). The overexpression lines showed 1.6-fold higher COMT enzyme activity than the wild-type, while the RNAi lines had 2.5-fold lower activity than the wild-type, suggesting that COMT activity was closely associated with ASMT enzyme activity in these transgenic lines (Fig. 9A). Finally, melatonin levels were measured to determine whether COMT expression is coupled with melatonin synthesis *in vivo*. As shown in Fig. 9B, melatonin levels were enhanced by more than two-fold on average in the overexpression lines compared with the wild-type, whereas the RNAi lines showed significantly reduced melatonin levels by more than two-fold compared with the wild-type controls. Taken together, the transgenic lines clearly showed that COMT expression plays a direct role in melatonin synthesis in plants and that its expression levels are closely coupled with melatonin levels.

## Discussion

The conversion of NAS into melatonin is the final step in melatonin biosynthesis and is catalysed by proteins with ASMT enzyme activity. The *ASMT* genes from plants were first cloned from rice (Park *et al.*, 2013a; Park *et al.*, 2013b), and an *ASMT* homologue was further cloned from apple (Zuo *et al.*, 2014). Although these *ASMT* genes encode enzymes with ASMT enzyme activity, the specific activity of purified recombinant ASMT was very low at 0.23 pkat mg protein^−1^ (Park *et al.*, 2013b). In addition, transgenic rice plants overexpressing these three *ASMT* genes showed only minor increases in ASMT enzyme activity (Park *et al.*, 2013a). Moreover, homologous *ASMT* genes were not found in dicotyledonous plants, such as *Arabidopsis*, which expresses a protein with 31% amino acid identity with rice ASMT1. It is unlikely that this *Arabidopsis* ASMT homologue (At4g35160) has ASMT activity, but this should be verified in future studies. Based on melatonin production in *Arabidopsis* (Gomez *et al.*, 2013; Shi and Chan, 2014; Lee *et al.*, 2015; Shi *et al.*, 2015), it is highly likely that an alternative *O*-methyltransferase with ASMT activity exists in *Arabidopsis* and other dicotyledonous plants.

Recently, an alternative *ASMT*-encoding gene was identified in *Arabidopsis*. Surprisingly, COMT, a well-known multifunctional enzyme responsible for lignin and flavonoid biosynthesis, shows ASMT enzyme activity (Byeon *et al.*, 2014a; Lee *et al.*, 2014). Although the *V*_max_ of AtCOMT for NAS conversion into melatonin was 313-fold lower than that for caffeic acid conversion into ferulic acid (Byeon *et al.*, 2014a), the relative ASMT activity (*V*_max_/*K*_m_) of OsCOMT was 609-fold higher than that of rice ASMT1 (Park *et al.*, 2013b). In addition to *V*_max_, the *K*_m_ values of AtCOMT and OsCOMT for NAS were 233 µM and 243 µM, respectively. These values were two-fold higher than that of caffeic acid (103 µM) (Byeon *et al.*, 2014a). In contrast, the *K*_m_ of ASMT for NAS was four-fold higher than that of COMT (Byeon *et al.*, 2014b), suggestive of higher NAS affinity for COMT rather than ASMT. Furthermore, as the physiological NAS concentration is as low as 0.1 nmol/g FW in rice (Park *et al.*, 2012), the conversion rate of NAS into melatonin by either COMT or ASMT was markedly attenuated and led, in part, to the very low level of melatonin synthesis in plants (Zohar *et al.*, 2011). The enzyme kinetic data suggest that COMT plays a significant role in melatonin biosynthesis in plants; however its specific role remains unclear. In this study, a direct role for OsCOMT in melatonin biosynthesis in rice plants was examined in 4-week-old detached leaves by measuring melatonin content. When the 4-week-old rice leaves were challenged with quercetin plus cadmium, melatonin synthesis was completely blocked, suggesting the involvement of COMT in cadmium-induced melatonin synthesis in rice plants (Fig. 6). In contrast to quercetin, caffeic acid did not result in a reduction in melatonin synthesis upon cadmium treatment. The precise reason for the inability of caffeic acid to inhibit melatonin synthesis in detached rice leaves remains unclear, but it may be attributed to ready oxidation of caffeic acid by interaction with oxidants or low mobility or low concentration of caffeic acid treatment in plant cells (Fig. 3). To examine the involvement of COMT in melatonin synthesis, transgenic rice plants with either overexpression or suppression of rice *COMT* were generated, and found that the level of *COMT* expression was closely related to ASMT enzyme activity and melatonin synthesis in these transgenic rice plants.

Although COMT is involved in the synthesis of melatonin *in vitro* and *in vivo* in rice plants, its participation is highly dependent on various substrates of COMT, which include caffeic acid, quercetin, 5-hydroxyferulic acid, 5-hydroxyconiferaldehyde, and coniferyl aldehyde (Koshiba *et al.*, 2013). These COMT substrates are intermediates in the biosynthesis of lignin, which accounts for 20–30% of plant biomass (Buchanan *et al.*, 2000). Lignin, flavonoid, and melatonin are all synthesized through the shikimic acid pathway, which is responsible for the synthesis of aromatic amino acids, such as tryptophan, tyrosine, and phenylalanine. Interestingly, 20% of the carbon fixed by plants enters this pathway, especially the lignin pathway. This suggests that lignin biosynthetic intermediates are abundant and widespread in plant tissues that efficiently suppress melatonin synthesis via COMT throughout the entire plant life cycle. In particular, some plants produce large amounts of flavonoids, such as quercetin (Miean and Mohamed, 2014), which may lead to depletion of melatonin. However, these quercetin- or phenolic-rich plants still produce a certain level of melatonin (Arnao, 2014; Feng *et al.*, 2014; Korkmaz *et al.*, 2014; Mukherjee *et al.*, 2014; Riga *et al.*, 2014; Tan *et al.*, 2014; Zhang *et al.*, 2014), suggesting a coordinated contribution of both COMT and ASMT in melatonin synthesis in plants. It is therefore highly likely that the inhibitory effects of quercetin and lignin intermediates, such as caffeic acid, on ASMT activity of COMT result in a predominant role for COMT in catalysis of lignin synthesis and not melatonin synthesis, because the levels of lignin intermediates were several orders of magnitude higher than those of NAS under conditions of normal plant growth (Koshiba *et al.*, 2013). However, these inhibitory effects of quercetin and caffeic acid are diminished under different growth conditions or stages of development, such as cadmium treatment, where COMT can play some role in melatonin synthesis. These types of interactions by COMT substrates may be partially responsible for variations in melatonin synthesis in plants (Zohar *et al.*, 2011; Tan *et al.*, 2014).

It is difficult to speculate on the rate-limiting step of melatonin synthesis in plants as multiple genes, including *SNAT*, *ASMT*, and *COMT*, are involved in last two stages of melatonin synthesis in plants. In addition, overexpression of *SNAT*, *ASMT*, or *COMT* in transgenic rice plants did not result in marked increases in melatonin synthesis, suggesting the possible irrelevance of these gene products as rate-limiting enzymes (Park *et al.*, 2013a; Byeon *et al.*, 2015a).

In summary, both *ASMT* and alternative *COMT* genes play pivotal roles in melatonin biosynthesis, and their possible coordinated expression may be involved in the unique temporal and spatial patterns of melatonin production, leading to pleiotropic functional roles of melatonin (Arnao and Hernández-Ruiz, 2014; Hardeland, 2015; Zhang *et al.*, 2015) in plant growth and development.
